# The effect of bowel preparation regime on interfraction rectal filling variation during image guided radiotherapy for prostate cancer

**DOI:** 10.1186/s13014-017-0787-y

**Published:** 2017-03-09

**Authors:** Ali Hosni, Tara Rosewall, Timothy Craig, Vickie Kong, Andrew Bayley, Alejandro Berlin, Robert Bristow, Charles Catton, Padraig Warde, Peter Chung

**Affiliations:** 1 Department of Radiation Oncology, Princess Margaret Cancer Centre/University of Toronto, 610 University Ave, Toronto, ON M5G 2M9 Canada; 2 Radiation Medicine Program, Princess Margaret Cancer Centre, Toronto, ON Canada

**Keywords:** Bowel preparation, Laxative, Prostate cancer, Radiotherapy, Rectum

## Abstract

**Background:**

This study aimed to investigate the tolerability and impact of milk of magnesia (MoM) on interfraction rectal filling during prostate cancer radiotherapy.

**Methods:**

Two groups were retrospectively identified, each consisting of 40 patients with prostate cancer treated with radiotherapy to prostate+/-seminal vesicles, with daily image-guidance in 78Gy/39fractions/8 weeks. The first-group followed anti-flatulence diet with MoM started 3-days prior to planning-CT and continued during radiotherapy, while the second-group followed the same anti-flatulence diet only. The rectum between upper and lower limit of the clinical target volume (CTV) was delineated on planning-CT and on weekly cone-beam-CT (CBCT). Rectal filling was assessed by measurement of anterio-posterior diameter of the rectum at the superior and mid levels of CTV, rectal volume (RV), and average cross-sectional rectal area (CSA; RV/length).

**Results:**

Overall 720 images (80 planning-CT and 640 CBCT images) from 80 patients were analyzed. Using linear mixed models, and after adjusting for baseline values at the time of planning-CT to test the differences in rectal dimensions between both groups over the 8-week treatment period, there were no significant differences in RV (*p* = 0.4), CSA (*p* = 0.5), anterio-posterior diameter of rectum at superior (*p* = 0.4) or mid level of CTV (*p* = 0.4). In the non-MoM group; 22.5% of patients had diarrhea compared to 60% in the MoM group, while 40% discontinued use of MoM by end of radiotherapy.

**Conclusion:**

The addition of MoM to antiflatulence diet did not reduce the interfraction variation in rectal filling but caused diarrhea in a substantial proportion of patients who then discontinued its use.

## Background

Advances in radiotherapy (RT) technology have permitted dose escalation in prostate cancer to improve biochemical control [1]. Precision of RT delivery is an essential component to improve outcomes and reduce associated treatment toxicity [2]. Prostate motion is mainly attributable to changes in rectal volume and shape [3, 4], this has led to various strategies to reproduce consistent rectal filling and provide increased accuracy of RT delivery for prostate cancer.

It has been suggested that using a rectal balloon to achieve reproducible large rectum is one way to reduce variations in rectal filling, thereby reducing prostate motion [5]. Other non-invasive strategies use a rectum-emptying approach, by means of laxatives, anti-flatulence diet [6], bowel relaxant [4], probiotics [7], enemas [8], rectum-emptying tube [9], self evacuation [10], or combination of these. However the degree of effectiveness of each of these methods and identification of the most successful approach is still debatable.

Since 1997, our institutional policy to reduce rectal variation consisted of a defined bowel regimen of an anti-flatulence diet and milk of magnesia (MoM). Nonetheless, subsequent studies using magnesium laxatives failed to show clinically relevant reduction of prostate motion, with high probability of less laxative intake in response to diarrhea [11–14]. Subsequently, our institutional practice changed in 2012 to simple dietary advice (anti-flatulence diet only) without the use of MoM. Although previous investigation had found no reduction in intrafraction prostate motion when using our bowel regimen (with MoM) [12], the efficacy for interfraction rectal filling was not evaluated. Therefore, in the present study we investigated the impact of MoM on the interfraction differences in rectal filling and assessed its tolerability.

## Methods

### Patient selection

Following institutional research ethics board approval, two sequential groups of localized prostate cancer patients treated with volumetric-modulated-arch-therapy (VMAT) to the prostate +/- seminal vesicles (SV) were retrospectively identified. Our institutional practice changed to simple dietary advice without the use of MoM in 2012, so each group consisted of randomly chosen 40 consecutive patients treated in 2011 (MoM cohort) and 2013 (non-MoM cohort). Exclusion criteria were: prostate cancer patients received palliative or postoperative radiotherapy or brachytherapy, pelvic lymph node involvement or distant metastasis, patients with inflammatory bowel disease or taking laxatives, stool softeners or anti-flatulence drugs for other indications.

### Bowel regimen

All patients participated in a routine educational session with a radiation therapist regarding the bladder and rectal preparation for radiotherapy planning and treatment. Patients in the MoM cohort received instructions to follow a bowel regimen which combined an anti-flatulence diet (Table 1) and MoM, while the non-MoM cohort followed the same anti-flatulence diet only. All patients were instructed to start the anti-flatulence diet +/- MoM three days before the planning CT scan and continue during RT. The initial once a day (bedtime) dose of MoM was 30 cm^3^, adjusted from 15 to 60 cm^3^ to achieve a soft bowel movement each morning and stopped in case of lower gastrointestinal (GI) toxicity (i.e. diarrhea). Bowel habit description and daily intake of MoM at baseline and weekly during RT were prospectively documented in the electronic medical record, as a standard of care. Lower GI toxicity (diarrhea during RT) was graded according to RTOG acute toxicity scoring criteria.

**Table 1:** Instructions for anti-flatulence diet

Types of food	Specific foods to avoid
Vegetables	Peas, beans, lentils, broccoli, cauliflower, brussel sprouts, cabbage, sauerkraut, cucumber, turnip, rutabaga, onions, garlic
Fruits	Apples, bananas, prunes, melons
High-fat foods	Pastries, pies, deep-fried foods
Carbonated drinks	Soda, beer

### Radiotherapy

Clinical target volume (CTV) included the prostate; while base of the SV was included in the CTV if the risk of SV involvement was > 15% [15]. Planning target volume (PTV) was created by expansion of the CTV by 10 mm in all directions, except 7 mm posteriorly. RT was delivered using VMAT to a prescribed dose of 78Gy in 39 fractions over 8 weeks, using daily prostate-focused image guidance with cone beam CT (CBCT). All patients were treated in the supine position, without rigid immobilization.

### Rectal motion assessment

For each patient, the outer rectal wall was delineated as a solid structure between the upper and lower limits of the CTV, as changes in rectal diameter at this level would likely have the greatest influence on prostate position. This was performed by a single observer on the planning CT and on eight randomly selected CBCTs (one from each week of RT). Rectal filling was assessed by measurement of the anterio-posterior diameter of the rectum at the superior and mid levels of CTV, and by calculation of rectal volume (RV) and the average cross-sectional rectal area (CSA; defined as the rectal volume divided by rectal craniocaudal length).

### Statistical analyses

Descriptive statistics were used to describe patient and treatment characteristics. Student’s *t*-test was used for comparison of continuous variables. Changes in anterio-posterior diameter of the rectum at the superior and mid levels of CTV, RV and CSA between the planning CT and weekly CBCT were compared between both groups by repeated measures analysis using linear mixed models. All tests were two-sided. Statistical analyses were performed using SAS system (version 9.4; SAS Institute Inc, Cary, NC).

## Results

### Patient characteristics

All 80 patients completed the intended course of RT as planned (78Gy over 39 fractions). The characteristics of the patients in both groups are summarized in Table 2. No patients received androgen deprivation treatment. The 640 CBCTs selected from the 80 patients were reviewed, and confirmed satisfactory visualisation of the bladder, prostate and seminal vesicles with good definition of rectal boundaries between the upper and lower levels of the CTV.

**Table 2:** Patient characteristics in MoM and non-MoM groups

	MoM group *N* = 40	Non-MoM group *N* = 40
Age (years)		
- Median	72	71
- Range	65 - 84	60 - 82
T-category		
- T1	22	24
- T2	18	16
Combined Gleason score		
- 6	4	5
- 7	36	35
PSA		
- Median	8	7
- Range	4 - 17	5 - 17
CTV volume (cm^3^)		
- Median	45	50
- Range	19 - 116	21 - 139

*MoM* milk of magnesia, *CTV* clinical target volume

### Interfraction rectal filling characteristics

In each group, a total of 360 images, including 40 planning CT and 320 CBCT images from the 40 patients were analyzed. Summary of descriptive statistics of rectal volume, average CSA, anterioposterior diameter of the rectum at superior and mid level of CTV in both cohorts at the time of planning CT are shown in Table 3.

**Table 3:** Descriptive statistics of rectal measurements at the time of planning

	MoM cohort	Non-MoM cohort	*p*-value
Mean rectal volume (cm^3^)	34.8 +/- 20.7	35 +/- 18.7	0.9
Mean average cross sectional area (cm^2^)	6.5 +/- 3.5	6.5 +/- 2.6	0.9
Mean anterioposterior diameter at superior level of CTV (cm)	3.2 +/- 1	3.2 +/- 0.8	0.9
Mean anterioposterior diameter at mid level of CTV (cm)	3.2 +/- 0.9	3 +/- 0.9	0.3

*MoM* milk of magnesia, *CTV* clinical target volume

**Fig. 1:**
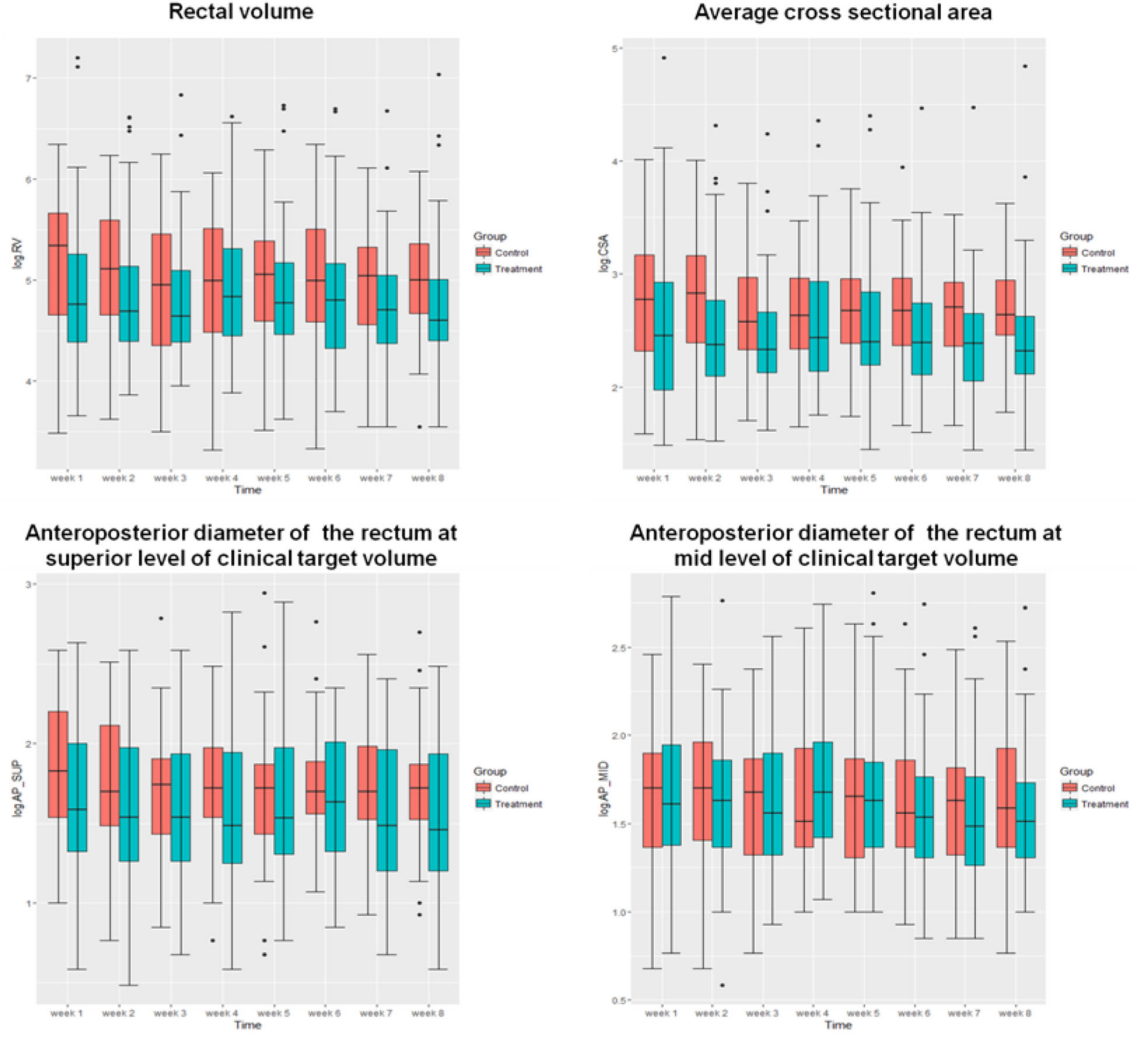
Interfraction rectal filling assessment in the MoM and non-MoM groups. *MoM*: milk of magnesia; *Control*: group of patients who didn't receive milk of magnesia; *Treatment*: group of patients who received milk of magnesia; *RV*: rectal volume; *CSA*: average cross sectional area; *AP_SUP*: Anterioposterior diameter of the rectum at the superior level of clinical target volume; *AP_MID*: Anterioposterior diamter of the rectum at the mid level of clinical target volume

The mean RV for MoM vs. non-MoM groups were 34.1+/- 21.9 vs. 35.5+/- 15.5 cm^3^, and the average CSA were 6.3+/-3.7 vs. 6.7+/-2.4 cm^2^, while the mean anterioposterior diameter of the rectum at superior and mid level of CTV were 3.2+/-1.1 vs. 3.4+/-1 cm and 3.2 +/-1 vs. 3.2+/-0.9 cm respectively. Using linear mixed models, and after adjusting for baseline values at the time of planning CT to test the differences in rectal dimensions between both groups over the 8-week treatment period, there were no significant differences between MoM vs. non-MoM group either for RV (*p* = 0.4), average CSA (*p* = 0.5), anterioposterior diameter of the rectum at superior level of CTV (*p* = 0.4) or anterioposterior diameter of rectum at mid level of CTV (*p* = 0.4) (Fig. 1).

### MoM tolerability and gastrointestinal toxicity

**Fig. 2:**
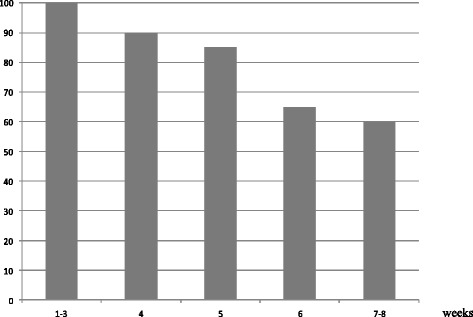
Proportion of patients who took milk of magnesia during radiotherapy

In the MoM group, the median volume of MoM taken by patients was 30 cm^3^ (range, 15–45 cm^3^) in the first week and 15 cm^3^ (range, 0–30 cm^3^) in the last week. The proportion of patients who took MoM decreased from 100% in the first week to 60% in the last week (Fig. 2). Acute RTOG lower GI toxicity in MoM vs. non-MoM groups consisted of G2 diarrhea in 3 patients (7.5%) vs. 2 patients (5%) and G1 diarrhea in 21 patients (52.5%) vs. 7 patients (17.5%). In both groups, the onset of diarrhea was reported in the second week of RT, however with higher probability among patients who took MoM (the number of patients who had G1 diarrhea in the second week of RT in MoM vs. non-MoM group was 9 [22.5%] vs. 5 [12.5%]).

## Discussion

This study demonstrated no significant difference between the MoM and non-MoM groups in the interfraction variability of rectal dimensions which could affect the prostate motion including RV, average CSA, anterioposterior diameter of the rectum at superior and mid level of CTV. Furthermore, G1-2 diarrhea was experienced in 24 (60%) patients in the MoM group compared to 9 (22.5%) patients who didn’t receive MoM, with 16 (40%) patients discontinuing the use of MoM by the end of radiation treatment.

Image guided radiotherapy (IGRT) is implemented to improve the accuracy of treatment delivery, however it remains difficult to correct for deformation and rotation of the prostate which is mostly influenced by changes in rectal filling. Previous studies have shown that changes in rectal filling can lead to poor outcomes following RT. [16, 17] Furthermore, maintaining consistent rectal filling leads to reduction of the required PTV margins [18], which enables dose escalation of RT to the prostate with probability of better tumour control [19], without increasing treatment toxicity thereby improving the therapeutic ratio.

The use of laxatives and anti-flatulence diet to reduce rectal filling variation has been previously investigated. In a randomised controlled trial (RCT) with 30 prostate cancer patients, Oat et al. reported that dietary intervention with psyllium (20 g/d, *n* = 15) didn't significantly reduce the variability in RV or rectal filling at superior level of the prostate. It was however, associated with consistent rectal filling at mid-level of the prostate [20]. In another RCT of prostate cancer patients assigned to receive magnesium oxide (500 mg twice a day, *n* = 46) or placebo (*n* = 46) during RT and similar to our results, there was no significant difference in RV between the treatment arms and magnesium oxide was not effective in reducing the interfraction rectal filling [14]. Furthermore, several other studies using magnesium laxatives were unable to show a clinically relevant reduction of inter- or intra-fractional prostate motion [11–13].

Despite an inability to reduce interfraction prostate motion, a dietary protocol with laxatives may potentially decrease the rectal distention related to gas resulting in better CBCT image quality and facilitating the IGRT process [11]. On the other hand, tolerability of the laxative remains an important clinical consideration, with higher probability of less laxative intake or even anti-diarrheal use in response to more frequent bowel movement or changing the stool texture [12, 13]. In a RCT of magnesium oxide (*n* = 46) vs. placebo (*n* = 46), patients in the intervention arm had more frequent grade ≥2 acute GI toxicity (37% vs. 22%) and were more often prescribed anti-diarrheal medicines during RT (15% vs. 9%) [13]. We had previously reported that the proportion of patients who didn’t take MoM increased from 8% in the first week to 44% in the last week of RT [12]. Consistently, in the current study, 60% of patients who took MoM had diarrhea, and 40% discontinued its use by the end of radiation treatment.

The findings from this study should be interpreted in context with its methodological limitations. The most significant limitation is related to its retrospective and non-randomized nature which may have led to undocumented differences between the cohorts which may have masked the effect of diet and MoM. Also, compliance with the antiflatulence diet was not quantified and may have been different between the two groups, which may have negated the effect of the diet on interfraction rectal filling variation. Nonetheless, when considered in context with previous evaluations of this subject, our results confirm that the addition of MoM to an antiflatulence diet does not lead to more consistent rectal filling. Variations in rectal volume and size can influence both translational prostate motion (which can be corrected with IGRT), and rotational prostate motion (which is more difficult to mitigate by current state-of-the-art technologies). However, rectal filling is not the sole factor with potential impact in prostate spatial localization, and different intervention approaches considering altogether the impact of rectal and bladder filling, patient positioning and breathing on prostate motion should be investigated.

## Conclusion

The addition of MoM to an antiflatulence diet did not reduce interfraction variation in rectal filling and may cause diarrhea resulting in a substantial proportion of patients discontinuing its use. Simple dietary instructions appeared to be just as effective at reducing interfraction rectal variability, and MoM should be omitted from routine use during prostate radiotherapy.

## Abbreviations

CBCT: Cone beam CT
CSA: Cross sectional area
CT: Computed tomography
CTV: Clinical target volume
GI: Gastrointestinal
IGRT: Image guided radiotherapy
MoM: Milk of magnesia
PTV: Planning target volume
RCT: Randomized controlled trial
RT: Radiotherapy
RV: Rectal volume
SV: Seminal vesicles
VMAT: Volumetric modulated arch therapy.
